# Clinical Outcomes in Patients With Left Bundle Branch Area Pacing vs. Right Ventricular Pacing for Atrioventricular Block

**DOI:** 10.3389/fcvm.2021.685253

**Published:** 2021-07-08

**Authors:** Xiaofei Li, Junmeng Zhang, Chunguang Qiu, Zhao Wang, Hui Li, Kunjing Pang, Yan Yao, Zhimin Liu, Ruiqin Xie, Yangxin Chen, Yongquan Wu, Xiaohan Fan

**Affiliations:** ^1^Department of Cardiology, Fuwai Hospital, National Center for Cardiovascular Diseases, Chinese Academy of Medical Sciences and Peking Union Medical College, Beijing, China; ^2^Department of Cardiology, Beijing Anzhen Hospital, Capital Medical University, Beijing, China; ^3^Department of Cardiology, the First Affiliated Hospital of Zhengzhou University, Zhengzhou, China; ^4^Department of Echocardiography, Fuwai Hospital, National Center for Cardiovascular Diseases, Chinese Academy of Medical Sciences and Peking Union Medical College, Beijing, China; ^5^Department of Cardiology, The Second Hospital of Hebei Medical University, Hebei Institute of Cardiovascular Research, Shijiazhuang, China; ^6^Department of Cardiology, Sun Yat-sen Memorial Hospital, Sun Yat-sen University, Guangzhou, China

**Keywords:** atrioventricular block, left bundle branch area pacing, heart failure hospitalization, upgrade to biventricular pacing, right ventricular pacing

## Abstract

**Background:** Left bundle branch area pacing (LBBAP) is a novel pacing modality with stable pacing parameters and a narrow-paced QRS duration. We compared heart failure (HF) hospitalization events and echocardiographic measures between LBBAP and right ventricular pacing (RVP) in patients with atrioventricular block (AVB).

**Methods and Results:** This multicenter observational study prospectively recruited consecutive AVB patients requiring ventricular pacing in five centers if they received LBBAP or RVP and had left ventricular ejection fraction (LVEF) >50%. Data on electrocardiogram, pacing parameters, echocardiographic measurements, device complications, and clinical outcomes were collected at baseline and during follow-up. The primary outcome was first episode hospitalization for HF or upgrade to biventricular pacing. LBBAP was successful in 235 of 246 patients (95.5%), while 120 patients received RVP. During a mean of 11.4 ± 2.7 months of follow-up, the ventricular pacing burden was comparable (83.9 ± 35.1 vs. 85.7 ± 30.0%), while the mean LVEF differed significantly (62.6 ± 4.6 vs. 57.8 ± 11.4%) between the LBBAP and RVP groups. Patients with LBBAP had significantly lower occurrences of HF hospitalization and upgrading to biventricular pacing than patients with RVP (2.6 vs. 10.8%, *P* <0.001), and differences in primary outcome between LBBAP and RVP were mainly observed in patients with ventricular pacing >40% or with baseline LVEF <60%. The primary outcome was independently associated with LBBAP (adjusted HR 0.14, 95% CI: 0.04–0.55), previous myocardial infarction (adjusted HR 6.82, 95% CI: 1.23–37.5), and baseline LVEF (adjusted HR 0.91, 95% CI: 0.86–0.96).

**Conclusion:** Permanent LBBAP might reduce the risk of HF hospitalization or upgrade to biventricular pacing compared with RVP in AVB patients requiring a high burden of ventricular pacing.

**Clinical Trial Registration:** URL: https://www.clinicaltrials.gov; Unique identifier: NCT03851315; URL: http://www.chictr.org.cn; Unique Identifier: ChiCTR2100043296.

**Graphical Abtract:**
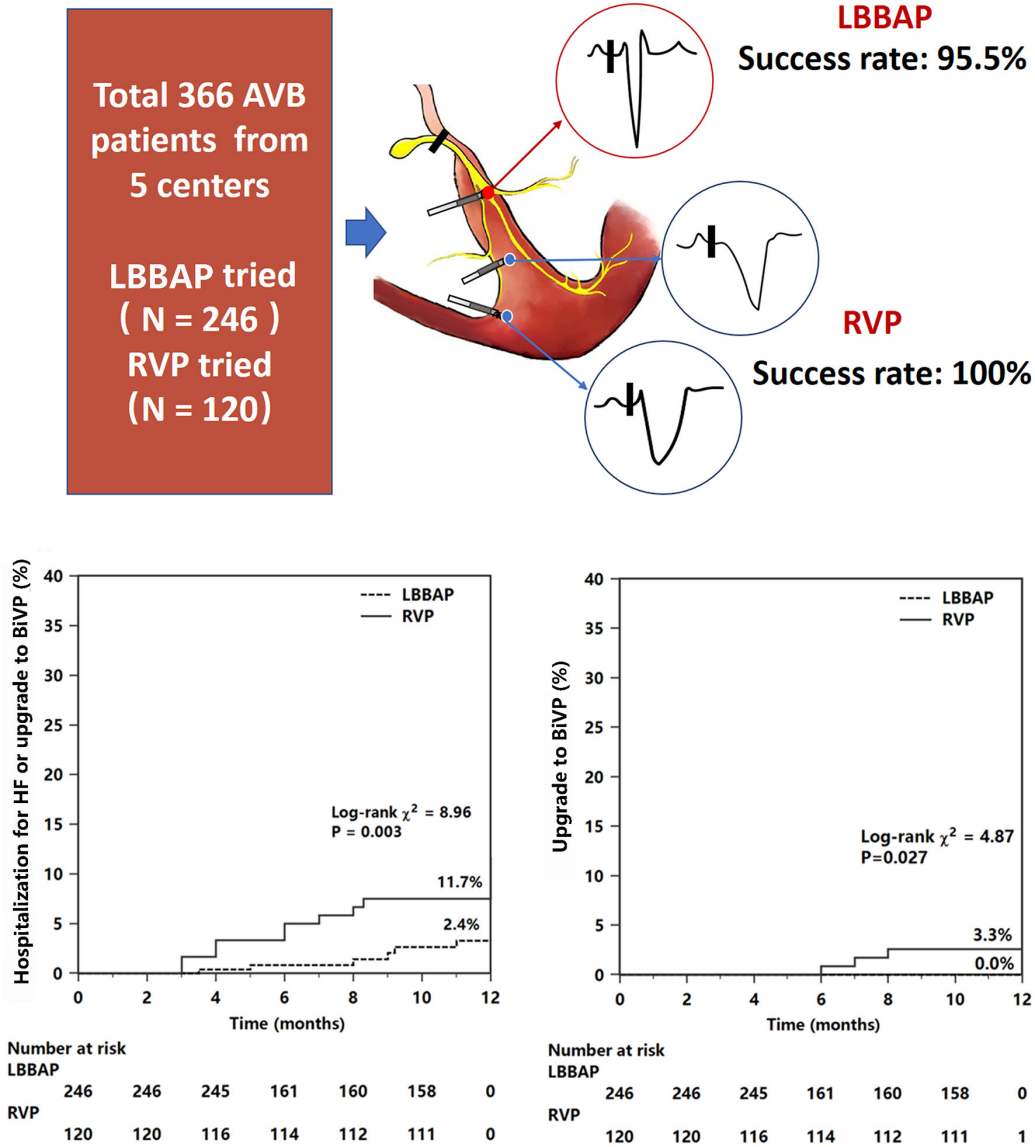
Comparison of clinical outcomes between LBBAP and RVP. The sketch has presented different pacing modes. Kaplan–Meier survival curves and analysis of the clinical outcomes in all patients. Figures and analysis show a statistically significant reduction in both the primary endpoint (composite endpoint of HF hospitalization or upgrade to BiVP) and upgrade to BiVP events associated with LBBAP compared with RVP. For abbreviations, see Figure 1.

## Introduction

Some patients with advanced atrioventricular block (AVB) may be at high risk of pacing-induced heart failure (HF) because conventional right ventricular pacing (RVP) can result in left ventricular mechanical dyssynchrony and impaired cardiac function (1). A previous study (2) reported that the risk of HF death was increased by 8% at every 10% increase in RVP burden. Biventricular pacing (BiVP) may prevent adverse left ventricular (LV) remodeling and a reduction in LV ejection fraction (LVEF) in bradycardia patients with normal systolic function (3) and reduce the progressive risk of HF in AVB patients with impaired cardiac function (4) compared with RVP. However, BiVP is not a routine treatment for AVB with preserved cardiac function due to the complicated procedure and expensive device. His bundle pacing can achieve normal paced QRS duration (QRSd) and ventricular mechanical synchrony (5). However, His bundle pacing is also not routinely used because of a low success rate, high risk of lead dislodgement, or raising threshold (5).

Left bundle branch area pacing (LBBAP) has emerged recently (6) as a new physiological pacing approach. LBBAP can achieve almost normal paced QRSd with a low and stable pacing threshold, good R wave sensing, and short procedure duration comparable to RVP (7–10). LBBAP can also correct bundle branch block (BBB) in bradycardia patients (11) and improve LV systolic function in patients with HF (12). However, most current studies focus on the feasibility, safety, pacing parameters, electrocardiogram, or echocardiographic features of LBBAP during short-term follow-up. The comparison of the long-term clinical effect on HF hospitalization events between LBBAP and RVP has not been reported in patients with AVB requiring a high burden of ventricular pacing (VP). This multicenter study aimed to prospectively observe HF hospitalization events between LBBAP and RVP in patients with AVB.

### Methods

This study was conducted at Fuwai Hospital, National Center for Cardiovascular Diseases, Beijing; Anzhen Hospital, Beijing; Sun Yat-sen Memorial Hospital, Guangzhou; the First Affiliated Hospital of Zhengzhou University; and the Second Hospital of Hebei Medical University. This prospective observational study was approved by the Institutional Review Board of all five hospitals in this study. All consecutive patients with AVB requiring ventricular pacing according to current guidelines were enrolled from 2019 if they signed written informed consent for an agreement of the implantation procedure and study analysis. Patients or the public were not involved in the design, conduct, reporting, or dissemination plans of our research.

### Study Population

**Figure 1:**
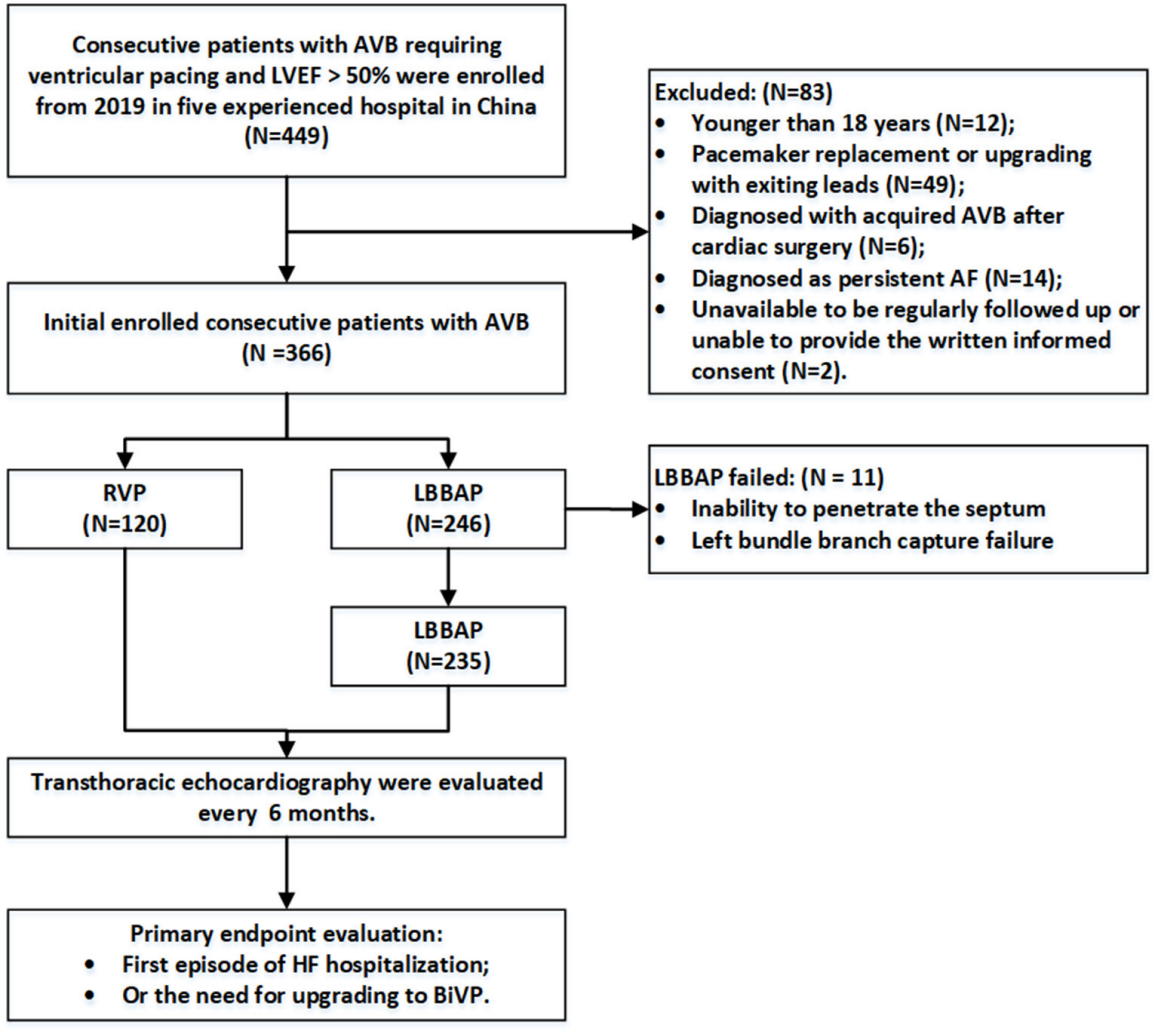
Flowchart of the study population enrollment. AVB, atrioventricular block; RVP, right ventricular pacing; LBBAP, left bundle branch area pacing; AF, atrial fibrillation; HF, heart failure; BiVP, biventricular pacing.

Patients with AVB recruited in this study were over 18 years old and had LVEF >50% at baseline. The pacing strategies were determined by operators as per the clinical practice at each hospital. The LBBAP group included all patients who attempted the LBBAP procedure, while the RVP group included patients undergoing RV apex or septum pacing. Patients were excluded if they (1) were younger than 18 years; (2) underwent pacemaker replacement or upgrading with existing leads; (3) had severe valvular diseases, congenital heart disease, or hypertrophic cardiomyopathy; (4) were diagnosed with acquired AVB after surgery for hypertrophic cardiomyopathy or other congenital heart diseases; (5) were diagnosed with persistent atrial fibrillation; and (6) were unavailable to be regularly followed up at the clinic visit for various reasons or to provide written informed consent (Figure 1).

### LBBAP Procedure

The LBBAP procedure has been previously described in detail (8). During the later period of the study, we simplified the implant procedure of the LBBAP ventricular pacing lead. Briefly, the Select Secure pacing lead of model 3830 (Medtronic, Inc., Minneapolis, MN, USA) was delivered through the C315 sheath (Medtronic, Inc.) after left axillary vein access. In the right anterior oblique 30° position, the sheath with a 3830 pacing lead was directly inserted into the right ventricle through the tricuspid annulus. The tip of the 3830 pacing lead was advanced slightly outside the sheath to touch the septum myocardium at an area 1.5~2.0 cm away from the tricuspid annulus. Unipolar pacing was performed at an output of 2.0 V/0.4 ms to identify a potential screwing site according to the following criteria: (1) a paced morphology of the QS complex with a notch in the bottom in lead V1 and (2) R wave amplitudes >5 mV. After screwing the lead deep into the septum, unipolar pacing was performed to assess the paced QRS morphology, the R wave amplitude, and pacing impedance. The stimulus-to-peak LV activation time (S-pLVAT) was measured at both low (2.0 V/0.4 ms) and high (5.0 V/0.4 ms) outputs in lead V_4−6_. Ring pacing was tested to evaluate the lead depth in the interventricular septum. Measures of impedance and R wave amplitudes are helpful to prevent lead penetration into the LV. If LBBAP could not be successful after five attempts, the lead was screwed into the interventricular septum to achieve deep LV septal pacing. The RV lead was implanted at the RV apex or septum by a shaping stylet to achieve stable pacing parameters.

Successful LBBAP was confirmed per the previously published criteria (8, 9, 11): (a) paced QRS morphology presented with an RBBB pattern and (b) S-pLVAT shortened abruptly and remained shortest and constant at different testing outputs. Selective LBBP was identified if a discrete component was presented between the spike and the QRS onset on intracardiac electrogram at a low output (usually at 0.5 V/0.4 ms), or left bundle branch potential could be recorded, or a transition in QRS morphology of V1 from “Qr” or “QR” type to “rsR” type with decreasing unipolar output could be observed. Sixty beats per minute with bipolar pacing mode was set in all of the patients. The pacing output was set as 3.0 V/0.4 ms at the first 3 months of follow-up. If the threshold remained stable at the 3-month follow-up, the automatic ventricular capture management algorithms might be turned on, or the pacing output would be set at 2.0–2.5 V/0.4 ms based on pacing thresholds. For patients with complete heart block, the atrioventricular delay was set as 150/120 ms after the procedure of both LBBAP and RVP. For patients with intermittent AVB, atrioventricular delay programming strategies were different between LBBAP and RVP. In patients with RVP, automatic atrioventricular delay optimization algorithms were routinely turned on to minimize the use of RVP. In patients with LBBAP, the atrioventricular delay was set 30 ms longer than the intrinsic atrioventricular interval if the patient had a normal intrinsic QRS duration. If patients had baseline bundle branch block, the atrioventricular delay was set 30 ms shorter than the intrinsic atrioventricular interval to achieve possible correction of electrical dyssynchronization.

### Clinical Outcomes and Follow-Up

The clinic visit follow-up was performed every 6 months after pacemaker implantation in each hospital. Echocardiographic evaluations were conducted at baseline, 6 months, and 1 year after the procedure by using Vivid E9 systems (GE Vingmed Ultrasound AS, Horten, Norway). Left ventricular end-diastolic diameter (LVEDD) and LVEF were evaluated by the core lab that was blinded to the pacing parameter settings, and in cases of disagreement, a senior echocardiographer was invited to read the original data to reach an agreement. Biplane Simpson's method in two-dimensional transthoracic echocardiography was used for the evaluation of LVEF.

The primary outcome was defined as a combined endpoint including the first episode of HF hospitalization or the need for upgrading to BiVP. The independent event committee adjudicated all events. HF hospitalization was identified if the patient presented to outpatients or emergency department visits or inpatient hospitalization with symptoms and signs consistent with HF and required diuretics and other therapy (vasodilation, etc.). The indications for requiring an upgrade to BiVP were according to current guidelines (13), including HF and AVB with reduced LVEF (<40%) after guideline-directed medical treatment for at least 3 months. The pacing parameters and ventricular pacing burden and 12-lead ECG were all recorded at baseline and at each follow-up visit. Lead-related complications were routinely tracked.

### Statistical Analysis

The statistical analyses were performed by SPSS version 24.0 (SPSS, Inc., Chicago, IL, USA) and GraphPad Prism 5 (GraphPad Software, Inc., San Diego, CA). Continuous variables were summarized using the means and standard deviation or median and interquartile range and compared with two-tailed Student's *t*-tests or Wilcoxon rank sum test. Nominal data are presented as frequencies and percentages and were compared by using the chi-square test or Fisher's exact test. Kaplan–Meier curves and univariate and multivariate Cox proportional hazard models were used to analyze the primary outcomes, and time censoring was determined by time to primary outcomes or time to last follow-up. All statistical tests were two-tailed. A *P* value of < 0.05 was considered significant.

## Results

### Baseline Clinical Characteristics and Implant Outcomes of Patients

A total of 366 consecutive patients were included. LBBAP was attempted in 246 patients, while 120 patients received RVP. As shown in Table 1, patients between the two groups had similar mean age, sex distribution, AVB grades, BBB types, and other clinical characteristics except for the prevalence of paroxysmal atrial fibrillation (29.3 vs. 15.8%, *P* = 0.005). Baseline LVEF was also comparable between the LBBAP (61.7 ± 7.4)% and RVP (61.5 ± 6.4)% groups.

**Table 1:** Baseline characteristics of AVB patients.

**Variables**	**LBBAP group (*N* = 246)**	**RVP group (*N* = 120)**	***P***
Age, years	63.3 ± 15	62.1 ± 17.2	0.575
Female, %	85 (34.6)	39 (32.5)	0.052
Paroxysmal atrial fibrillation, %	72 (29.3)	19 (15.8)	0.005
Hypertension, %	132 (53.7)	65 (54.2)	0.927
Diabetes, %	50 (20.3)	25 (20.8)	0.910
Coronary arterial disease, %	31 (12.6)	20 (16.7)	0.292
MI history, %	11 (4.5)	4 (3.3)	0.606
Dilated cardiomyopathy, %	6 (2.4)	0 (0.0)	0.085
Valvular heart disease, %	19 (7.7)	12 (10)	0.463
Baseline QRSd	115.9 ± 26.7	117.9 ± 27.9	0.514
**Conduction disorders**			
Marked first-degree AVB, %	20 (8.1)	8 (6.7)	0.621
Second-degree AVB, %	59 (24.0)	27 (22.5)	0.753
High-grade AVB, %	47 (18.9)	24 (20.0)	0.839
Third-degree AVB, %	120 (48.8)	61 (50.8)	0.712
AVB with sinus node dysfunction, %	71 (28.9)	30 (25.0)	0.438
Left bundle branch block, %	37 (17.9)	15 (14.2)	0.402
Right bundle branch block, %	59 (28.5)	33 (31.1)	0.629
**Echo data**			
Baseline LVEDD, mm	49.4 ± 6.6	49.6 ± 5.9	0.787
Baseline LVEF, %	61.7 ± 7.4	61.5 ± 6.4	0.738
NT-proBNP, pg/ml	261.5 (89.3, 864.3)	424.2 (100.1, 976.7)	0.301

*MI, myocardial infarction; LVEDD, left ventricular end-diastolic diameter; LVEF, left ventricular ejection fraction*.

**Figure 2:**
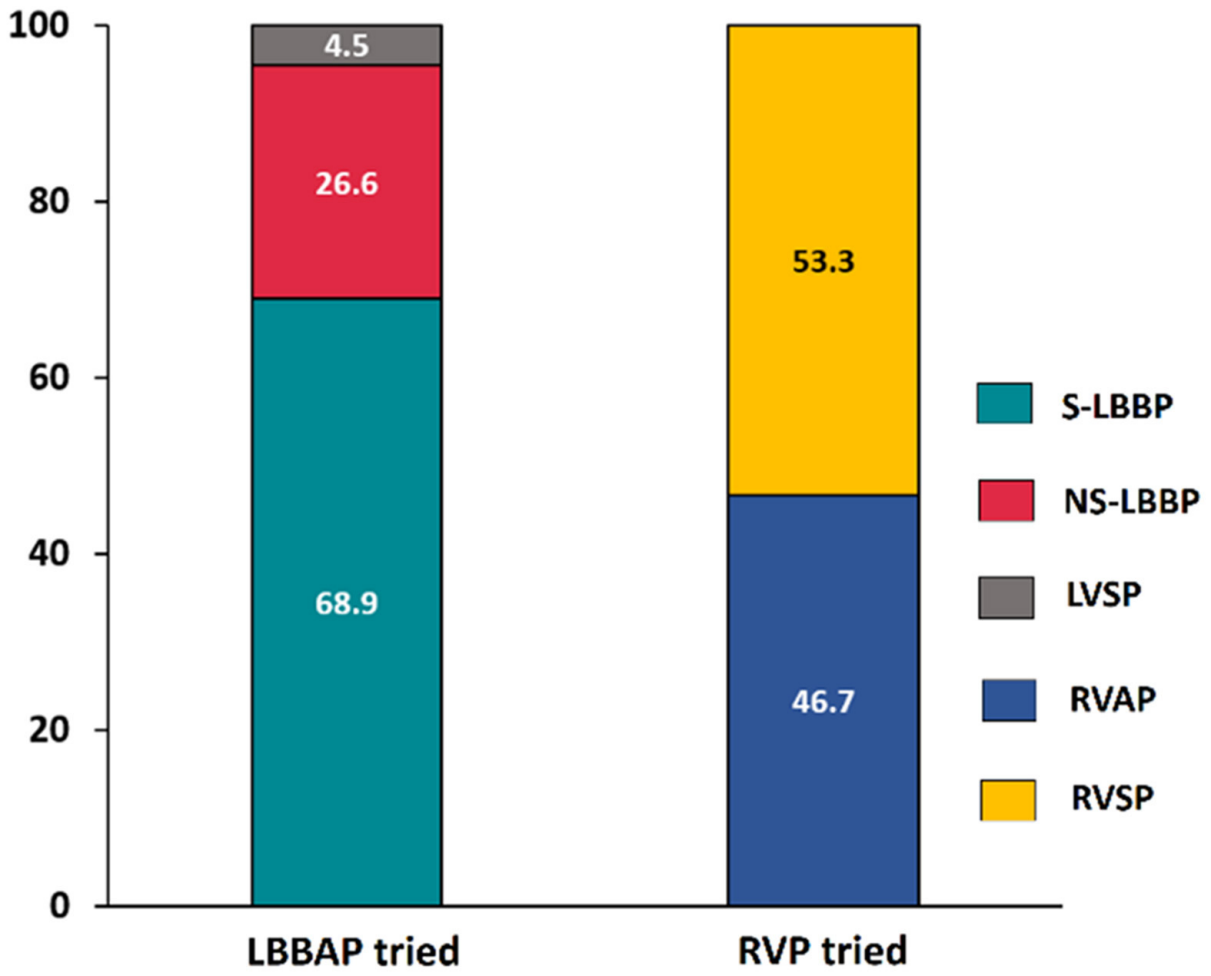
Comparison of success rate between LBBAP and RVP. LBBAP, left bundle branch area pacing; S-LBBP, selective left bundle branch pacing; NS-LBBP, non-selective left bundle branch pacing; LVSP, left ventricular septal pacing; RVAP, right ventricular apical pacing; RVSP, right ventricular septal pacing.

Permanent LBBAP was successful in 235 of 246 patients (95.5%), with selective LBBP in 162 (68.9%) patients. As shown in Figure 2, deep LV septal pacing was performed in 11 patients. The reasons for LBBAP failure included the inability to penetrate the septum in five patients and failure to capture the left bundle branch in six patients. In the RVP group, there were 56 (46.7%) apical pacing and 64 (53.3%) septal pacing.

### Pacing Parameters and Procedure Complications During Follow-Up

The mean follow-up duration was 11.4 ± 2.7 months. Table 2 shows the pacing parameters and complications during the procedure and follow-up. Compared with RVP, LBBAP showed better sensing R wave amplitude, lower pacing impedance, and similar pacing threshold and significantly narrower QRSd during the procedure and at the 6-month follow-up. The ventricular pacing percentage was comparable between these two groups (83.9 ± 35.1 vs. 85.7 ± 30.0%, *P* = 0.614). At the 1-year follow-up, the pacing threshold and sensing R wave amplitude were comparable between the two groups. The lower pacing impedance and narrower QRSd (112.3 ± 16.3 vs. 152.9 ± 40.8 ms, *P* < 0.001) remained in the LBBAP group.

**Table 2:** Pacing characteristics during the procedure and follow-up.

**Variables**	**LBBAP (*N* = 235)**	**RVP (*N* = 120)**	***P***
Dual-chamber pacemaker	235 (100)	120 (100)	1.000
**During the procedure**			
Sense, mV	12.4 ± 11.2	9.6 ± 5.7	0.013
Threshold, V/0.4 ms	0.67 ± 0.23	0.66 ± 0.24	0.762
Impedance, Ω	757.2 ± 164.0	853.6 ± 258.5	<0.001
Paced QRSd, ms	114.2 ± 13.8	158.5 ± 25.5	<0.001
**6-month follow-up**			
Sense, mV	14.9 ± 5.4	11.7 ± 5.6	<0.001
Threshold, V/0.4 ms	0.73 ± 0.25	0.65 ± 0.67	0.122
Impedance, Ω	577.1 ± 145.7	647.8 ± 184.0	<0.001
Paced QRSd, ms	112.5 ± 15.3	153.5 ± 32.6	<0.001
VP, %	83.9 ± 35.1	85.7 ± 30.0	0.614
**1-year follow-up**	***N*** **=** **173**	***N*** **=** **109**	
Sense, mV	14.8 ± 4.8	13.0 ± 3.6	0.213
Threshold, V/0.4 ms	0.8 ± 0.3	0.7 ± 0.2	0.180
Impedance, Ω	621.3 ± 149.0	771.2 ± 184.4	0.002
Paced QRSd, ms	112.3 ± 16.3	152.9 ± 40.8	<0.001
**Complications**			
Septal perforation during the procedure	5 (2.1)	0 (0.0)	0.172
Septal or apical perforation after procedure	1 (0.4)	1 (0.8)	0.668
Dislocation during follow-up	1 (0.4)	3 (2.5)	0.114

*VP, ventricular pacing percentage*.

The complications in the LBBAP group were similar to those in the RVP group. Even though five patients (2.1%) suffered septal perforation during the procedure, the perforation did not cause any symptoms. Only one septal perforation occurred 2 h after the procedure and resulted in dislodgement and ventricular capture failure. After repositioning the pacing lead, most patients underwent successful LBBAP with uneventful recovery. Lead perforations or dislodgement was not found following hospital discharge. In the RVP group, apical perforation occurred in one patient, ventricular lead dislocation occurred in three patients during follow-up, and all patients underwent uneventful lead revision.

### Comparison of Echocardiographic Measures

Compared with baseline, patients with LBBAP had stable LVEF and slightly decreased LVEDD at the 1-year follow-up (Table 3). In contrast, patients with RVP had gradually decreased LVEF and significantly increased LVEDD from baseline to 6 months and at 1-year follow-up. The comparison between RVP and LBBAP at 1-year follow-up showed a significant difference in LVEF (62.6 ± 4.6 vs. 57.8 ± 11.4%, *P* = 0.004) and LVEDD (46.6 ± 5.2 vs. 51.7 ± 7.5 mm, *P* = 0.005).

**Table 3:** Echocardiographic measures at baseline and during follow-up.

**Variables**	**LBBAP (*N* = 235)**	**RVP (*N* = 120)**	***P***
**Baseline**
LVEDD, mm	49.4 ± 6.6	49.6 ± 5.9	0.787
LVEF, %	61.7 ± 7.4	61.5 ± 6.4	0.738
**6-month follow-up**
LVEDD, mm	48.4 ± 6.5	49.4 ± 6.5	0.435
LVEF, %	61.2 ± 6.7	58.6 ± 9.4^*^	0.045
**One-year follow-up**
LVEDD, mm	46.6 ± 5.2^*^	51.7 ± 7.5^*^	0.005
LVEF, %	62.6 ± 4.6	57.8 ± 11.4^*^	0.004

* *Compared with baseline status, P < 0.05*.

*LVEDD, left ventricular end-diastolic diameter; LVEF, left ventricular ejection fraction*.

### Clinical Outcomes

**Figure 3:**
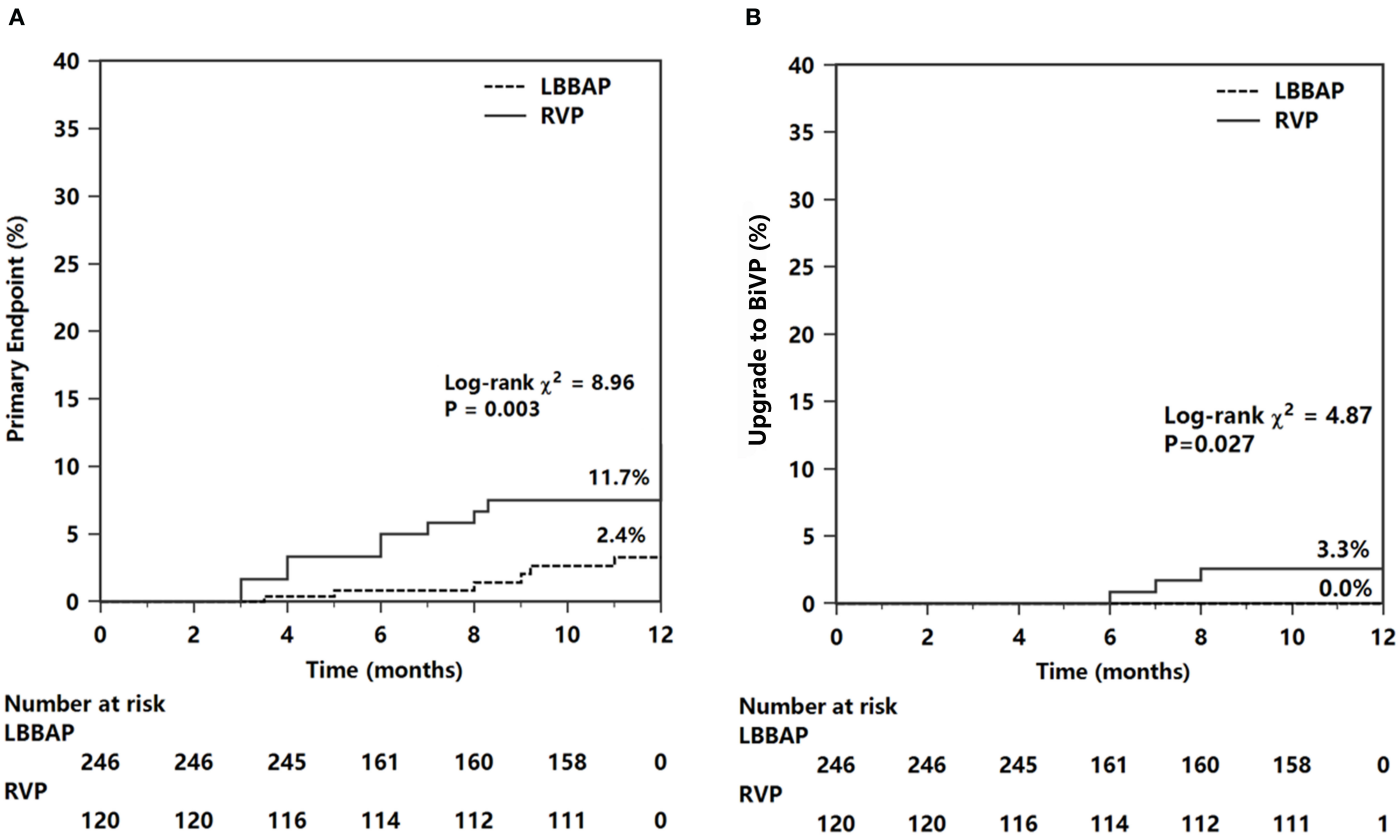
Kaplan–Meier survival curves and analysis of the primary endpoint **(A)** and endpoint of upgrade to BiVP **(B)**. For abbreviations, see Figure 1.

The primary composite endpoint of HF hospitalization and upgrading to BiVP was 2.6% in the LBBAP group and 10.8% in the RVP group (*P* <0.001, Table 4). Among patients who suffered HF hospitalization, upgrading to BiVP occurred in four patients in the RVP group during three to nine months of follow-up compared with zero patients in the LBBAP group. The Kaplan–Meier analysis showed a trend toward higher HF hospitalization (*P* = 0.003) and a higher occurrence of an upgrade to BiVP (*P* = 0.027) in the RVP group than in the LBBAP group (Figure 3). In Table 5, the univariate and multivariate Cox analyses showed that the LBBAP pacing modality was an independent predictor for a reduced risk of the primary composite outcome (adjusted HR 0.14, 95% CI: 0.04–0.55, *P* = 0.005). HF hospitalization and upgrading to BiVP were also associated with a history of previous myocardial infarction (adjusted HR 6.82, 95% CI: 1.23–37.75, *P* = 0.028) and LVEF at baseline (adjusted HR 0.91, 95% CI: 0.86–0.96, *P* = 0.001).

**Table 4:** Clinical outcomes evaluation.

**Variables**	**LBBAP (*N* = 235)**	**RVP (*N* = 120)**	***P***
HF hospitalization, *N* (%)	6 (2.6)	13 (10.8)	<0.001
Upgrade to BiVP, *N* (%)	0 (0.0)	4 (3.3)	0.011

*HF, heart failure; BiVP, biventricular pacing*.

**Table 5:** Univariate and multivariate Cox analyses for the composite outcome of HFH or upgrading to BiVP.

**Variables**	**Univariate analysis**			**Multivariate analysis**		
	**HR**	**95% CI**	***P***	**Adjusted HR**	**95% CI**	***P***
LBBAP vs. RVP	0.25	0.09–0.71	0.009	0.14	0.04–0.55	0.005
Female	0.64	0.25–1.62	0.686			
Age, years	0.99	0.96–1.02	0.455			
Atrial fibrillation	1.46	0.51–4.15	0.479			
Coronary arterial disease	1.39	0.40–4.84	0.605			
MI history	3.52	0.80–15.39	0.095	6.82	1.23–37.75	0.028
Dilated cardiomyopathy	4.06	0.54–30.60	0.174			
Valvular heart disease	0.04	0.01–81.65	0.415			
HCM (post Morrow)	1.97	0.26–14.79	0.514			
Baseline QRSd	1.01	0.99–1.02	0.582			
Baseline LVEF	0.93	0.90–0.97	<0.001	0.91	0.86–0.96	0.001
VP	1.02	1.00–1.05	0.087			

*LBBAP, left bundle branch area pacing; RVP, right ventricular pacing; CAD, coronary atrial disease; MI, myocardial infarction; DCM, dilated cardiomyopathy; VHD, valvular heart disease; HCM, hypertrophic cardiomyopathy; LVEF, left ventricular ejection fraction; VP, ventricular pacing percentage*.

**Figure 4:**
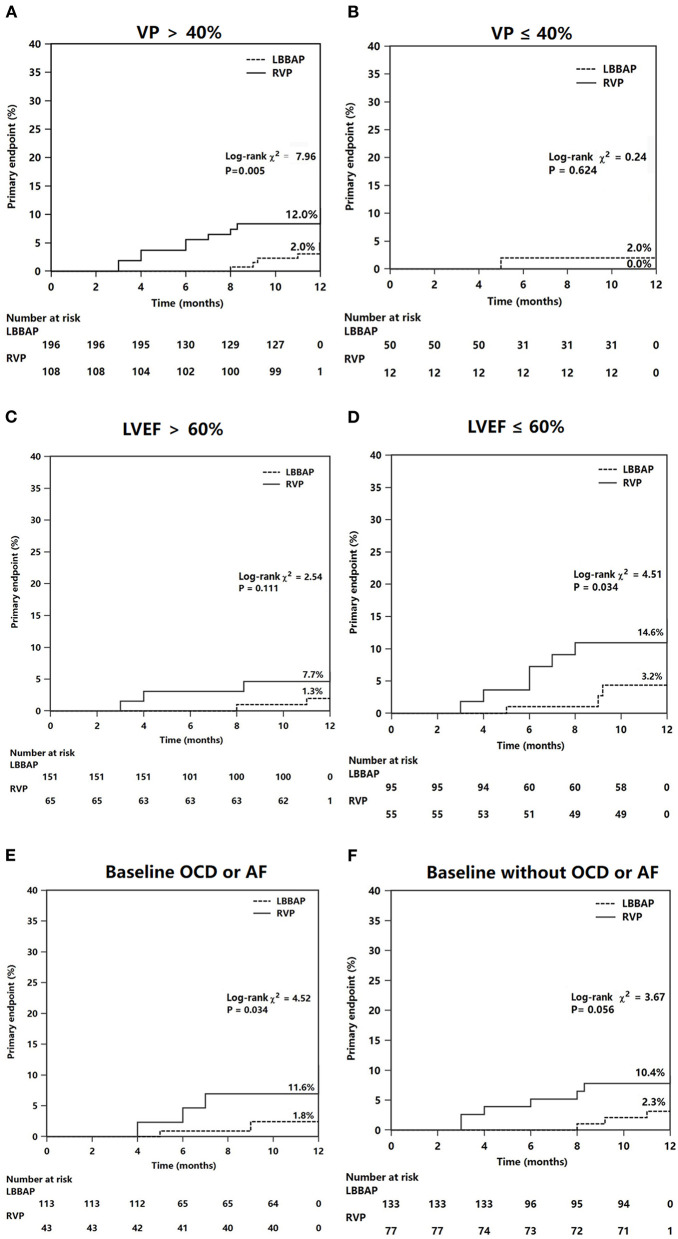
Subgroup Kaplan–Meier survival curves and analysis of the primary endpoints. The composite heart failure events was analyzed according to different groups of VP **(A,B)**, LVEF **(C,D)**, and status of OCD or AF at baseline **(E,F)**. VP, ventricular pacing (percentage); OCD, organic cardiac disease; AF, atrial fibrillation.

The results of the subgroup analysis are shown in Figure 4. The significant reduction in composite HF hospitalization events associated with LBBAP was confirmed in patients with VP burden >40% (2.0 vs. 12.0%, *P* = 0.005) (Figure 4A) but not in patients with VP burden ≤ 40% (Figure 4B). The difference in composite HF events did not statistically differ between RVP and LBBAP in patients with LVEF >60% (Figure 4C). However, in patients with baseline LVEF <60% (*n* = 150), the RVP group had significantly higher composite HF events than the LBBAP group (14.6 vs. 3.2%, *P* = 0.034) (Figure 4D). In patients with baseline organic cardiac disease (coronary artery disease, old myocardial infarction, mild dilated cardiomyopathy, or valvular heart disease) or atrial fibrillation, the primary HF events differed significantly between the LBBAP and RVP groups (1.8 vs. 11.6%, *P* = 0.034, Figure 4E). The trend toward a reduction in the primary outcome in patients with LBBAP compared with RVP did not reach statistical significance (2.3 vs. 10.4%, *P* = 0.056, Figure 4F) in patients without baseline organic cardiac disease or atrial fibrillation.

## Discussion

This multicenter prospective study demonstrated that permanent LBBAP presented stable pacing parameters and procedural complications similar to RVP during a 1-year follow-up. In patients with normal cardiac function and a high burden of VP, LBBAP achieved preserved LVEF and reduced LVEDD, while RVP resulted in reduced LVEF and enlarged LVEDD. Patients with LBBAP had a significant reduction in HF hospitalization events (including upgrading to BiVP) compared with conventional RVP (central illustration). The effect of LBBAP was seen predominantly in patients with VP >40%, patients with LVEF ≤ 60%, or patients with baseline organic cardiac disease or AF. LBBAP was an independent predictor for a reduced risk of HF hospitalization after adjustment for other risk factors.

The detrimental effect of traditional RVP has been associated with an increased risk for HF hospitalization and mortality in patients with a high VP burden (14). A recent study (15) indicated that an RVP > 20% is an independent risk factor for pacing-induced cardiomyopathy in AVB patients with baseline preserved LV function during a mean follow-up of 4.3 years. His bundle pacing is effective in preventing ventricular dyssynchrony and can reduce the risk of death, HF hospitalization, or upgrading to BiVP by 35% in patients with a VP burden of >20% (16). Consistent with previous studies (15), we found that HF hospitalization or upgrading to BiVP was common in patients with VP > 40%, baseline 50% < LVEF ≤ 60%, or baseline organic cardiac disease or AF. Pacing-induced HF hospitalization has been reported to occur within the first 6 months (17). The high RVP% in our study (85.7%) might be the main contributor to the occurrence of HF hospitalization events. In addition, damage to the myocardium (previous myocardial infarction) and mildly reduced baseline cardiac function (50% < LVEF ≤ 60%) might be the underlying reasons for the increased risk of HF hospitalization.

LBBAP can pace the conduction system beyond pathological or disease-vulnerable regions to produce nearly physiological ventricular capture. In recent studies, LBBAP generally achieves paced QRSd within 130 ms, mostly between 110 and 120 ms (7–11, 18–20). This study verified the narrower-paced QRSd by LBBAP at the 1-year follow-up in patients with a high VP burden. Because the capture thresholds of His bundle pacing might be unstable and increase during long-term follow-up (21), the long-term stability of low pacing thresholds of LBBAP has been questioned. A previous study (22) reported comparable R wave amplitudes and pacing thresholds between LBBAP and RVP at the 6-month follow-up. Our study confirmed the low and stable pacing thresholds of LBBAP at the 1-year follow-up and similar sensing amplitudes to those of RVP in patients with AVB and a high burden of VP. Although our previous study showed similar success rates of LBBAP (91.3%) to His bundle pacing (87.2%) (23), successful LBBAP appears to be easily achieved with increasing procedure experience. Huang et al. (24) reported a high success rate of LBBAP (97.8%) in their single-center experience, while a comparable success rate of LBBAP (93%) was reported by Vijayaraman et al. (9). The success rate of LBBAP in the present study (95.5%) was slightly higher than that (90.9%) in our previous study (8) due to increasing procedure volume and experience.

LBBAP could achieve LV synchrony and preserve LV function in bradycardia patients with normal cardiac function (8). A recent study (25) evaluated the systolic dyssynchrony index and the standard deviation of time-to-peak contraction velocity in LV 12 segments among native-conduction mode, LBBAP, and RVP situations and found that the LV synchrony of LBBAP is similar to that of native-conduction mode and superior to that of RV septal pacing. LBBAP could correct left bundle branch block (LBBB) and deliver cardiac resynchronization therapy (CRT) to effectively improve LV function and reduce HF symptoms in patients with HF and LBBB (18). In several small sample sizes of studies with mid-term follow-up (12, 26, 27), the effect of LBBAP on LV systolic function and CRT response appears to be superior to that of BiVP-delivered CRT. In addition, successful LBBAP can shorten QRS duration in bradycardia patients with right bundle branch block (RBBB) (9, 11, 28).

To our knowledge, this is the first study comparing LV function and clinical outcomes between LBBAP and RVP in AVB patients with high VP burden and baseline narrow QRSd. The significant difference in LVEF and LVEDD between the LBBAP and RVP groups at 1-year follow-up verified the beneficial effect of LBBAP on cardiac function. Although patients with normal cardiac function usually have few clinical outcomes after receiving RVP, our study still observed a significant difference in HF hospitalization events between LBBAP and RVP. A high burden of VP > 40% has been recognized as a risk factor for HF events during long-term follow-up. Our subgroup results further indicated that LBBAP might provide an additional benefit of cardiac function in patients with VP > 40%, baseline decreased LVEF (<60%), or baseline organic cardiac disease or atrial fibrillation. This is also the first study reporting the occurrence of HF hospitalization in patients with a high burden of LBBAP. Six patients in the LBBAP group presented HF symptoms and relatively reduced LVEF (>50 and <60%), and they recovered well after receiving medical treatment, including oral diuretics and beta-blockers. No patients in the LBBAP group presented with indications for upgrading to BiVP. Our results together with previous studies indicate that LBBAP might effectively reduce the risk of HF hospitalization compared with RVP in patients with normal LV function and a high burden of VP.

## Study Limitation

The main limitation of this study is the observational study design. The clinical homogeneity of patients could not be guaranteed between LBBAP and RVP. However, the higher prevalence of atrial fibrillation in the LBBAP group further demonstrated the potential benefit of LBBAP compared with RVP. Second, the relatively small sample size and the high percentage of RVAP in the RVP group might contribute to the difference in the clinical outcomes between RVP and LBBAP. Third, the clinical outcomes of all-cause death or cardiovascular death during longer follow-up may provide more solid evidence for the superiority of LBBAP. Therefore, future prospective randomized clinical trials with a large sample size are needed in patients with a high burden of VP.

## Conclusion

The results of this multicenter observational study indicate that LBBAP might be a preferable pacing modality to reduce potential HF events in patients requiring a high burden of VP compared with traditional RVP.

## Data Availability Statement

The raw data supporting the conclusions of this article will be made available by the authors, without undue reservation.

## Ethics Statement

The studies involving human participants were reviewed and approved by Institutional Review Board of Fuwai Hospital, Beijing; Institutional Review Board of Anzhen Hospital, Beijing; Institutional Review Board ofSun Yat-sen Memorial Hospital, Guangzhou; Institutional Review Board of the First Affiliated Hospital of Zhengzhou University, Zhengzhou and Institutional Review Board of the Second Hospital of Hebei Medical University, Hebei, China. The patients/participants provided their written informed consent to participate in this study.

## Author Contributions

XF, XL, YC, and YW conceptualized and designed the research. XF, XL, JZ, CQ, HL, KP, ZW, ZL, and RX were responsible for the acquisition, analysis, and interpretation of data. XF had obtained funding and supervised the work. XF and XL drafted the manuscript. YY, YC, and YW critically revised the manuscript. All authors contributed to the article and approved the submitted version.

## Conflict of Interest

The authors declare that the research was conducted in the absence of any commercial or financial relationships that could be construed as a potential conflict of interest.
